# Ambient Assisted Living as Support for Aging in Place: Quantitative Users’ Acceptance Study on Ultrasonic Whistles

**DOI:** 10.2196/11825

**Published:** 2018-12-12

**Authors:** Hannah Biermann, Julia Offermann-van Heek, Simon Himmel, Martina Ziefle

**Affiliations:** 1 Chair of Communication Science Human-Computer Interaction Center Rheinisch-Westfälische Technische Hochschule Aachen University Aachen Germany

**Keywords:** ambient assisted living, technology acceptance, user diversity, ultrasonic whistles, aging in place

## Abstract

**Background:**

Given the fact of an aging society, new supply measures and living concepts are needed, especially as health impairments along with care dependency increase with age. As many elderly people wish to stay at home for as long as possible, ambient assisted living (AAL) represents a support for aging in place.

**Objective:**

AAL combines medical and care technology within living environments and is, therefore, a promising approach to cope with demographic change in terms of fast-growing care needs and fewer skilled workers. Ultrasonic whistles represent one innovative technical possibility for such supportive housing solutions. Central fields of application are home automation, emergency service, and positioning. As AAL technologies affect sensitive areas of life, it is of great interest under which conditions they are accepted or rejected, taking individual user requirements into account. Hence, the aim of this study was to investigate users’ perception and evaluation of ultrasonic whistles.

**Methods:**

In this study, we examined the acceptance of ultrasonic whistles in home care by function and room using a Web-based questionnaire. Besides an evaluation of the overall usefulness, we focused on the intention to use ultrasonic whistles; 270 participants assessed home automation, emergency service, and positioning as specific functions of ultrasonic whistles. Furthermore, bathroom, bedroom, and living room were evaluated as specific usage locations (rooms). With regard to the user’s perspective, the focus was set on age and attitudes toward aging of care receivers.

**Results:**

This study revealed a significant influence of function (*F*_2,269_=60.444; *P*<.001), room (*F*_2,269_=41.388; *P*<.001), and the interaction of function and room (*F*_4,269_=8.701; *P*<.001) on the acceptance of ultrasonic whistles. The use of emergency services within the bathroom represented the most accepted alternative, whereas positioning within the living room received the comparably lowest evaluations. Although user diversity played a minor role for acceptance overall, the assessment of single applications differed among user groups, particularly with regard to age differences (*F*_20,500_=1.988; *P*<.01) in the evaluation of specific installation options such as automated doors.

**Conclusions:**

The study revealed profound insights into the user-centered assessment of ultrasonic whistles in home care and discovered function and room as influencing acceptance parameters. Concerning user characteristics, age, and attitude toward aging partly affected these evaluations, forming the basis for and showing the importance of further investigations in this context.

## Introduction

### Background

Against the background of demographic change, today’s society is affected by global shifts in population structure [1]. In Germany, for example, less young and a rising number of elderly people pose serious challenges, especially for health care [2]. Although better living conditions and medical progress extend life expectancy, the risk for health impairments increases with age [2]. In addition, chronic diseases (eg, diabetes) place new demands on the health care system [3]. Thus, nursing services face a growing amount of care-dependent people leading to shortcomings in the care supply [4]. As a consequence, it is observable that the majority of people in need of care are supplied with health services provided by relatives at home [5]. Hence, concepts referring to aging in place have become more important [6].

In this context, ambient assisted living (AAL) technologies and systems offer great potentials to simplify and enhance everyday life for both sides, patient and (family) caregiver, by means of technology assistance [7]. This refers to either individual devices (eg, stair lifts) or integrated systems (such as fall alarm systems). In addition, considering that many elderly people do not want to move out of their home [8], AAL technologies provide reliable prevention and rehabilitation measures to live independently despite physical or mental restrictions [7]. According to this, ultrasonic whistles—a specific technology to be used in supportive housing solutions (smart homes)—come up with new technical possibilities to support aging in place that are flexible in their application, retrofittable, cost-effective, and eco-friendly. The final ultrasonic whistle will be tiny and, thus, can be fitted in various places within the living environment such as furniture, textiles, or portable equipment on the body. Realizing an interconnectivity of infrastructure, major functions are home automation (eg, automated doors), safety prevention (eg, emergency services), and positioning (eg, fall detection) [9,10]. The handling is kept simple and also easy to use for people with disabilities, for example, by pressing a button. Technically, acoustic signals (ultrasound) are generated by actuation, coded by frequency, and inaudible to human ears. Referring to the durability of the ultrasonic whistle, first prototypes made of stiff plastic were quite damageable and sensitive when used in real-life scenarios. The air reservoir was frail toward fingernails when pushed from aside, the material got weak after several hundred times of pressing, and the reservoir got stuck down. In addition, the individual pressure strength and velocity lead to different frequencies. Using silicone as a softer raw material and adding a spring mechanism solved all problems toward reliability and durability; however, this prototype got a little bigger and needs to be scaled down for above-mentioned use cases. The decoding was taken over by receiving devices, installed in ceiling lights, for example, which activate the intended function and if required, forward data to third parties such as family caregivers, nursing services, or emergency centers. One major advantage of using this placing spot is that nearly all existing and new planned properties have ceiling lights; hence, you have electricity at a central point of each room. Concerning the microphones and receiving technology, first prototypes used a 4-channel ultrasonic sensor wired to a personal computer via ethernet for real-time data processing, gaining a robust algorithm to detect ultrasonic whistles. Last prototypes were implemented in embedded systems into multisensors in the ceiling. Transmission for existing projects can be realized through the very robust DECT (wireless connection) standard or for new projects using Konnex (KNX) wiring (a standard for building automation), which can be connected to uninterruptible power supply systems, which are standard in hospitals or case-sensitive buildings.

As AAL technologies are used in sensible and intimate home care contexts, their acceptance is not given without restrictions and highly dependent on individual requirements, for example, related to needs for safety, privacy, and autonomy but also to the general willingness of individuals to accept technology at home. Thus, it needs to be considered to what extent user diversity gains an impact on AAL acceptance [11]. In this context, prior user studies examined decisive influencing factors, for instance, gender [12], age [13,14], and experience with care [15]. Provided that elderly people wish to decide on their living situation [8], it is of great interest under which conditions they are (not) willing to accept the implementation of AAL technologies for specific contexts of use. Concerning home care, previous research showed that technology types and installation sites affect AAL acceptance [16,17].

To the best of the authors’ knowledge, there has been no empirical study directed to the acceptance of ultrasonic whistles in home care so far. Thus, this study aimed to examine the user’s acceptance of ultrasonic whistles, with special regard to their functions and usage locations (rooms). With regard to the importance of users’ diversity, we particularly focused on health status, user experience with (AAL) technologies, gender, age, and aging, and related their impact on the willingness to use AAL technologies.

### Ambient Assisted Living for Aging in Place

In this section, AAL is described as a chance for aging in place, with special attention given to ultrasonic whistles as an innovative technology. According to the current development of demographic change, growing potentials of AAL technologies in an aging society are outlined first. Thereafter, we discuss existing technology acceptance models, particularly focusing on a lack of knowledge referring to the influence of user diversity on the acceptance of specific AAL technologies. In this context, the focus is set on the factors of age and attitudes toward aging in relation to AAL acceptance.

### Ambient Assisted Living Potentials in an Aging Society

In Germany, demographic change causes an aging society combined with a declining population [18]. By 2060, the predicted number of inhabitants will range between 67.6 million and 73.1 million (depending on the scale of net immigration), provided that 1 out of 3 will be aged 65 years or older and the number of 70-year-olds will be almost twice of the newborns [2]. This shift in the age structure is because of persistently low birth rates and increasing life expectancy because of improved infrastructure and medical technological progress [18]. Aging involves the risk of health impairments such as physical or mental diseases (eg, cardiac insufficiency and dementia). Hence, the growth of an aging society poses major challenges, especially affecting the health care sector. Although the need for long-term care increases sharply beyond the age of 75 years [18], nursing staff decreases, as there are fewer people in working age resulting in a lack of care [4]. In 2015, more than two-thirds (2.08 million) of all German care-dependent people (2.9 million) received domiciliary care, provided that the majority (1.38 million) was cared for exclusively by family caregivers [5].

AAL as a support for aging in place offers a great potential to enable frail and elderly people to handle daily life autonomously and relieve caregivers [7]. In general, aging in place is defined as “the ability to live in one’s own home and community safely, independently, and comfortably, regardless of age, income, or ability level” [19]. Next to individual perceptions of life satisfaction [20], well-being [21], and senses [8], research focus is set on supportive housing solutions [22], with special regard to smart homes [23]. Smart homes are commonly understood as “digital environments that are sensitive, adaptive, and responsive to human needs” [24]. According to this, they are particularly suited for AAL providing reliable measures aimed at prevention, rehabilitation, and practical support in everyday life by means of technology assistance [25]. Apart from single technologies (eg, blood pressure monitors and wheelchairs), intelligent (smart) home environments are provided with software for multipurpose usage that is integrated unobtrusively into the living space to operate infrastructure [26]. On the basis of the idea of ubiquitous computing [27], smart homes support daily life by monitoring users’ behavior, technically realized through wireless interconnectivity (eg, sensor technologies for home automation and fall detection) and operated via user-friendly devices that facilitate natural communication and interaction through speech, gestures, and familiar interfaces, for instance, mobile phones [7]. Major applications for home care are medical monitoring and rehabilitation (eg, medication reminder and vital parameter monitoring), safety prevention (eg, floor sensors for fall detection), and home automation (eg, automatic lighting) [28-31].

In that respect, ultrasonic whistles could offer innovative technical possibilities for supportive housing solutions. They are small and unobtrusive and can be integrated into a variety of home locations. Next to fixed and local installations (eg, wall switches), it is possible to integrate ultrasonic whistles into wearable devices (eg, emergency call wristband). Respective designs are variable too and can be used to prevent stigmatization, as for instance, covert and aesthetic installations with regard to (younger) users who are still mobile and able to move actively. However, accessibility—especially for users with physical or motoric disabilities—can be guaranteed on demand in terms of coarse buttons, for example, that are easy to use and robust without risks of injury. As it is possible to place the ultrasonic whistle in different spots within immediate reach, depending on users’ requirements and their living environment, its application—including emergency situations— is reliable and safe. As ultrasonic whistles are a holistic assistance system, they can be flexibly adapted to individual user’s needs such as the automation of household tasks, safety, and fall prevention, as well as movement detection. In its specific function, the ultrasound is generated mechanically (eg, via button press), without using electricity or batteries. Hence, when using ultrasonic whistles, energy is produced in an environmentally friendly way, which represents a key feature [10], especially compared with equivalent assistance systems such as more complex and into-the-floor integrated sensor systems. However, there is only little knowledge about the user-centered perception and acceptance of ultrasonic whistles with regard to their technical possibilities in home care.

### Technology Acceptance, Ambient Assisted Living, and User Diversity

Previous research in the field of perception and acceptance of AAL technologies and systems revealed mainly positive evaluations by diverse user groups with regard to age, aging, and experience with disabilities [15,32,33]. At the center of those studies, perceived benefits in terms of a more independent, autonomous, and longer life at the own home environment contrast with perceived barriers, for example, feelings of surveillance, a perceived invasion of privacy, as well as feelings of isolation [34-36]. In particular, numerous qualitative studies (eg, interviews [32] and focus groups [17,37]) explored perceptions of AAL technologies in people older than 60 years. As key results, the older participants valued the opportunity given by AAL of staying longer at their own home, they understood the crisis in care (lack of caregivers and increasing proportions of people in need of care), as well as the potential of AAL technologies to relieve people in need of care, their caregivers, and the care sector itself. However, they also expressed concerns as they feared a dependency on not easy to control technologies, an invasion of privacy by storage or transfer of personal data, and a substitution of human caregivers by technology. Besides qualitative investigations, these motives and barriers have been confirmed by numerous quantitative surveys [15,16]. Predominately, generic AAL systems have been investigated so far (mostly not specific systems nor specific functions). However, there is a lack of knowledge about the interplay of specific AAL functions and application areas in home care and its importance for AAL acceptance.

For investigating the acceptance of assisting information and communication technologies and AAL technologies, well-known and widely spread acceptance models such as technology acceptance model [38], unified theory of acceptance and use of technology [39], and their adapted versions were frequently used in the past years. These models (in particular mentioned in David’s study [38]) are useful to predict the acceptance of innovative technologies by key constructs such as the perceived ease of use and the intention to use a specific technology. Those models are, however, quite generic and do not cover different contexts of use, usage situations, or specific user groups. Hence, the model has to be extended and specifically tailored to the ultrasonic whistle technology and their functions. For the application here, we added the functions (contrasting home automation, emergency service, and positioning) and the application areas (bathroom, bedroom, and living room) based on a preceding qualitative study to the ease-of-use and intention-to-use dimensions. In addition, previous studies and models showed the importance of integrating demographic user factors and individual attitudes into technology acceptance research, in general and in research referring to AAL technology acceptance, in particular (eg, [39] and AAL-related research: eg, [15,40]).

### Age, Aging, and Their Impact on Ambient Assisted Living Acceptance

As the risk for health impairments increase with age [18], elderly people represent a major user group concerning AAL. Although AAL technologies facilitate everyday life offering great potentials for aging in place, their acceptance is not given without restrictions and is dependent on individual requirements and the extent to which innovative technology is perceived useful [14,16]. This is especially the case in older adults, which is a highly heterogeneous user group, with special regard to biological age, technical generations, and attitudes toward aging [13,41-44].

In general, there is evidence that the willingness to use AAL is high, refuting widespread stereotypes against technophobia among seniors [13,41,42]. However, it is also recognized that older users show comparatively low levels of expertise and confidence when dealing with technology, which can be explained against their generational background [42,43]. Hence, elderly people rather hesitate to adopt innovative technology compared with younger groups [45]. In addition, age-related motoric and cognitive impairments can make it difficult to handle technology and, thus, may limit its acceptance.

Recent studies indicate a link between attitudes toward aging (eg, related to quality of life, social integration, active aging, and dealing with change) and AAL acceptance, provided that people with positive aging concepts are more willing to accept AAL technologies than people with negative attitudes toward aging [40,44]. This leads to the assumption that individual attitudes and perceptions are at least as important as demographic factors in this context. Hence, it is worth considering whether and to what extent a connection between age, aging, and AAL acceptance exists. Concerning the adoption of innovative technology, special attention is required toward personal characteristics and requirements to reduce potential barriers for greater access and usage at older age.

## Methods

### Aim of Study and Research Questions

The aim of this study was to validate the influence of individual factors on the acceptance of ultrasonic whistles in home care, with special regard to function and room. Considering user diversity, we focused on age and attitudes toward aging in relation to AAL acceptance based on previously conducted interview studies [40]. To provide statistical evidence in this context, a quantitative online questionnaire study was conducted addressing the following research questions (RQ):

*RQ1:* In regard to which functions, contrasting home automation, emergency service, and positioning, is the use of ultrasonic whistles accepted or rejected?

*RQ2:* In regard to which rooms, contrasting bathroom, bedroom, and living room, is the use of ultrasonic whistles accepted or rejected?

*RQ3:* Do user diversity factors, in particular age and attitudes toward aging but also gender, affect the assessment of ultrasonic whistles with regard to function and room?

The questionnaire was conducted as an open survey in Germany in summer 2017. Concerning the sample construction, the focus was set on three different age groups, with special regard to young (≤40 years), middle-aged (41-70 years), and elderly (≥71 years) people as they may differ in their perception and acceptance of innovative technology against generation differences. Participation was voluntary and anonymous. The participants were acquired by personal contact, email, and social media without payment and provided with a link to access the questionnaire. To reach a broad sample, particularly with regard to elderly people, paper-and-pencil questionnaires were additionally used. Thereafter, the questionnaire design was outlined in detail, before a sample description was given.

### Questionnaire Design

**Figure 1:**
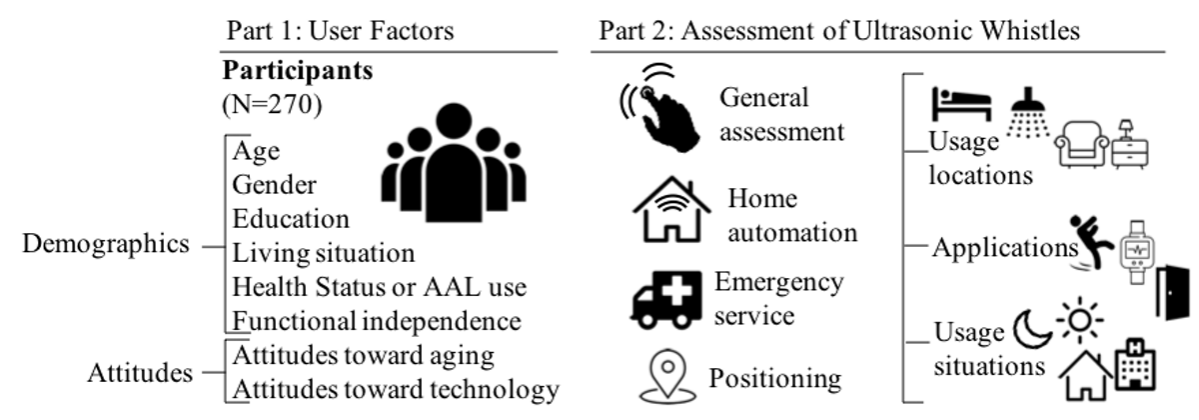
The illustration shows the questionnaire’s structure divided into 2 parts: user factors (left) and the assessment of ultrasonic whistles (right). AAL: ambient assisted living.

The questionnaire items were based on literature review according to the current state of research and prestudy results [40]. Overall, the questionnaire covered 33 questions within different sections (illustrated in Figure 1).

Initially, demographic data were collected such as age, gender, education, living situation, and health status. In connection with that, data about the current use of AAL technologies were collected, particularly with regard to blood pressure monitor, emergency service, bath lift, wheelchair, and motion sensor (answer options: yes or no). Subsequently, functional independence questions [46] were asked concerning the ability to handle activities of daily living, especially self-care, sphincter control, mobility, locomotion, communication, and social cognition (6 items; 7-point Likert scale with *min*=1: “in complete need of assistance” and *max*=7: “full autonomy”; Cronbach alpha=.907 by deleting the item *social cognition*).

Thereafter, the participants were asked to evaluate attitudes toward aging referring to the categories of health, dealing with change, active aging, social integration, and autonomy [40] on a 6-point Likert scale (*min*=1: “strongly disagree” and *max*=6: “strongly agree”). In total, 10 items (Cronbach alpha=.807; n=259) were used to measure positive (4 items) and negative (6 items) attitudes toward aging (see Textbox 1).

In addition, the participants were asked to assess their attitude toward technology (ATT) [48], such as technical experience, interest, and trust when dealing with technology (5 items; 6-point Likert scale with *min*=1: “strongly disagree” and *max*=6: “strongly agree”; Cronbach alpha=.899).

The acceptance of ultrasonic whistles in home care was evaluated within four sections structured as follows: (1) the overall use of ultrasonic whistles and the assessment focused on specific functions, namely, (2) home automation, (3) emergency service, and (4) positioning, with special regard to different usage locations, which were bathroom, bedroom, and living room.

**Categories and items (translated from German) referring to attitudes toward aging. Items measuring negative attitudes toward aging are coded reverse (R).**

Health

- Decreasing health (R)
- Being less fit and lively (R) [47]

Dealing with change

- Being more relaxed
- Less enjoyment (R) [47]

Active aging

- Making plans
- Keep on learning [47]

Social integration

- Staying in contact
- Loneliness (R) [47]

Autonomy

- Being a burden to others
- Being dependent (R) [40]

Overall, the assessment followed a two-step procedure within each section (1 to 4, provided that the sequence of sections was randomized). First, an evaluation of the perceived usefulness according to the rooms in question (bathroom, bedroom, and living room) was conducted using emoticons on a symbolic scale (3 items per section; see Table 1). Thereafter, the participants were asked to assess the respective use intention on a 6-point Likert scale (3 items per section, with all over Cronbach alpha>.9; see Table 1).

In addition, specific applications were evaluated on 6-point Likert scales (*min*=1: “strongly disagree” and *max*=6: “strongly agree”) such as applications at doors for home automation, wearables for emergency service, and floor applications for positioning (16 items in total, with all over Cronbach alpha>.9; see Textbox 2).

At the end of each section, the participants were faced with a final decision concerning the acceptance or rejection of ultrasonic whistles with regard to different usage situations (see Textbox 3), measured on a 6-point Likert scale (*min*=1: “strongly disagree” and *max*=6: “strongly agree”; 3 items per section, with all over Cronbach alpha<.7 in sections 1 to 3 and alpha=.740 in section 4).

Overall, mean values above the scale center (mean>3.5) indicated acceptance, whereas mean values below the average (mean<3.5) were interpreted as rejection.

**Table 1:** Evaluation of ultrasonic whistles by function and room referring to their perceived usefulness and use intention. For the perceived usefulness, we used symbolic answering patterns (smileys indicating rejection, abstention, or acceptance). For the use intention, we used 6-point Likert scales (min=1 “strongly disagree” and max=6 “strongly agree”).

Assessment sections	Perceived usefulness	Use intention
Ultrasonic whistle	I consider ultrasonic whistles as useful in bathroom/bedroom/living room.	I would use ultrasonic whistles in bathroom/bedroom/living room.
Home automation	I consider home automation as useful in bathroom/bedroom/living room.	I would use home automation in bathroom/bedroom/living room.
Emergency service	I consider emergency service as useful in bathroom/bedroom/living room.	I would use emergency service in bathroom/bedroom/living room.
Positioning	I consider positioning as useful in bathroom/bedroom/living room.	I would use positioning in bathroom/bedroom/living room.

**Evaluation of ultrasonic whistles referring to specific installations and conditions.**

I would use ultrasonic whistles

… at doors

… at windows

… in cupboards

… at chairs

… in wall switches

… in floor mats

I would wear ultrasonic whistles in emergency call wristbands

… during the day

… at night

… when I am alone

… at any time

I would use ultrasonic whistles in floors for fall detection

… in floor mats (detection)

… in doorsills

… in floorboards or tiles

… in carpets

… in selected parts of the dwelling

… throughout the home

**Evaluation of ultrasonic whistles referring to different usage situations.**

I would use ultrasonic whistles/home automation/emergency service/positioning

… in personal care situations

… for the care of relatives

… at the present time

**Figure 2:**
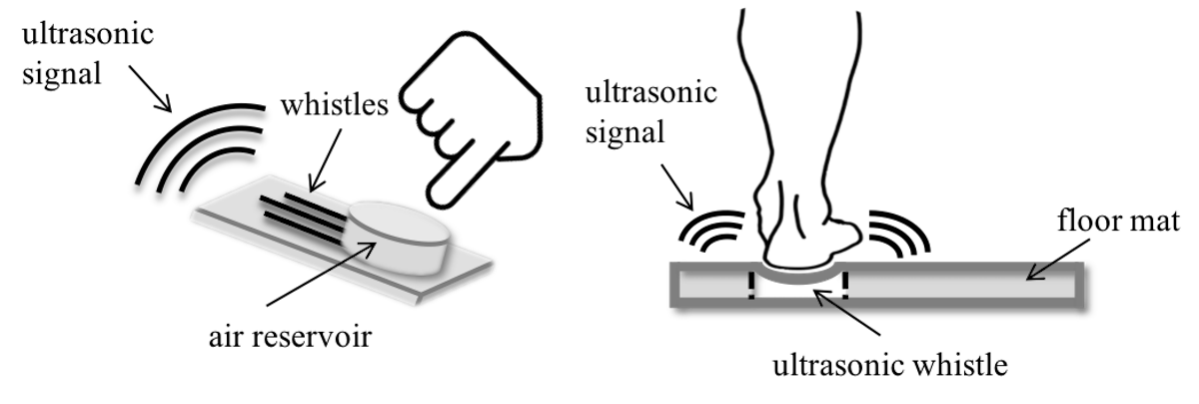
The illustration demonstrates the use of ultrasonic whistles by touch as for emergency service (left) and positioning (right).

### Instruction

As ultrasonic whistles represent an innovative assistance system, the participants were informed about technical core features and application options in advance. To reach a high understandability and to provide an idea of the functions of ultrasonic whistles, a drawing of the exemplary usage situations of ultrasonic whistles was given, see Figure 2: emergency buttons (left) and sensitive floors for fall detection (right, based on [9]). In addition, we provided a short scenario to ensure that all participants had the same level of knowledge in this context:

Imagine that either you or relatives are in need of home care due to health restrictions. Ultrasonic whistles make it possible to life actively and autonomously, though. Its implementation is unobtrusive. As ultrasonic signals are generated by touch, no electrical power is needed. Based on diverse whistle lengths, each ultrasonic whistle produces an individual signal, which is transmitted by air towards a receiver (e.g., installed in ceiling lights) to activate a specific function defined in advance, such as automatic door opening or further home automated tasks, emergency calls to relatives or nursing services, and positioning to detect danger and fall situations.

Pretests with participants in diverse ages ensured an overall understanding of the material and a maximum response time of 20 min with regard to all target groups.

### Sample Description

**Figure 3:**
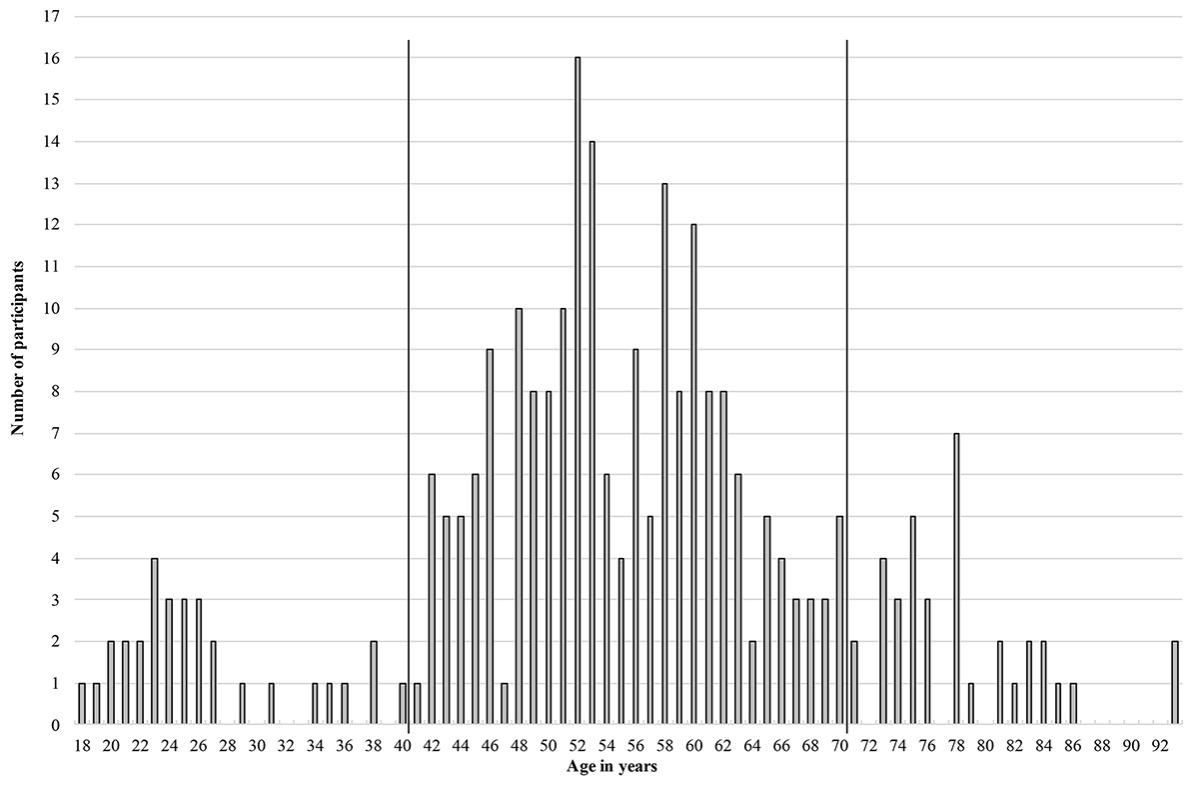
Age distribution ranging from 18 to 93 years with separation in age groups.

A total of 354 people participated in the questionnaire study. Of these, 84 had to be excluded from statistical analyses because of incomplete datasets. In all, a sample of 270 remained, of which 164 participants were female (164/270, 60.7%) and 106 were male (106/270, 39.3%). As the population share of women increases with age, especially concerning people aged 60 years and older [18], gender distribution can be explained in terms of demographic change. The participants’ age ranged from 18 to 93 years, with an average of 54.7 years (SD 14.67). According to the distribution (see Figure 3), we formed three age groups that showed peaks corresponding to the current age structure of Germany’s population [49].

With 42.2% (114/270) holding a university degree, the participants were educated above average [50]. Regarding their housing situation, most of the participants (188/270, 69.9%) lived in residential communities.

The majority (251/270, 92.9%) indicated that they wanted to stay at home for as long as possible at older age. The overall health status was good; 34.1% (92/270) were affected by chronic diseases, 18.5% (50/270) reported current health issues, and 5.2% (14/270) were care dependent with the help of family caregivers or nursing services, for example. Functional independence was high (mean 6.86 [SD 0.65]) and the use of AAL technologies was rather low (86/270, 31.9%). Overall, blood pressure monitors were most commonly used (137/270, 50.7%), followed by motion sensors (43/270, 15.9%), wheelchairs (13/270, 4.8%), emergency services (10/270, 3.7%), and bath lifts (4/270, 1.5%). In general, attitudes toward technology (mean 4.55 [SD 1.00]) and aging (mean 4.1 [SD 0.71]) were positive.

Correlation analyses (see Table 2) revealed significant relations between attitude toward aging and functional independence (*r*_*s*_=.138; *P*<.05). In addition, a relation between ATT and functional independence was observable (*r*_*s*_=.129; *P*<.05). Besides, age correlated with ATT (*r*_*s*_=−.227; *P*<.001) and functional independence (*r*_*s*_=−.216; *P*<.001). Interestingly, age and attitude toward aging were not related (*r*_*s*_=.005; *P*=.94); thus, older adults did not report different attitudes toward aging in comparison with younger adults.

**Table 2:** Correlations of demographic and individual user factors.

User factors	Age	Attitude toward aging	Attitude toward technology	Functional independence
Age	—^a^	.005	−.227^b^	−.216^b^
Attitude toward aging	—	—	.092	.138^c^
Attitude toward technology	—	—	—	.129^c^
Functional independence	—	—	—	—

^a^Not applicable.

^b^*P*<.001.

^c^*P*<.05.

### Aging and Its Characteristics

Overall, aging has to be understood as a complex factor, not only because the population share of people in later age is steadily rising but also as the onset of aging and the forms and extent vary interindividually [11]. For the analysis of user factors, we compared young, middle-aged, and elderly age groups (see Table 3). Hence, younger users (mean 4.92 [SD 1.01]) shared rather positive ATT compared with middle-aged (mean 4.61 [SD 0.93]) and elderly people (mean 3.89 [SD 1.14]), which can be explained with regard to different technology generations [51]. Concerning this study’s sample, the group of middle-aged people (born between 1947 and 1976) primarily experienced household and computer technologies, whereas the group of younger adults (born between 1977 and 1999) grew up with internet and social media as a part of the internet generation and so-called digital natives. By contrast, the group of elderly people (born between 1924 and 1946) gained comparatively low experiences when dealing with technology because of the historical circumstances of their past.

Moreover, people with positive attitudes toward aging, who were optimistic about life quality in later age along with possibilities for living independently, active aging, fairly well health, and social inclusion, slightly tended to state more positive attitudes toward technology (mean 4.62 [SD 0.90]) compared with the group of participants with negative aging concepts (mean 4.29 [SD 1.29]), who were concerned about social isolation, dependency on others, restrictions on life quality, and reduced mobility because of physical and mental diseases (see Table 4).

Hence, it is not only important to explore single user factors but, in fact, to discover their interaction effects with regard to different contexts of use.

**Table 3:** Descriptive profiles of young (≤40 years), middle-aged (41-70 years), and elderly (≥71 years) age groups.

User factors		Young (n=31)	Middle-aged (n=203)	Elderly (n=36)
Age (years), mean (SD)		26.29 (6.00)	54.85 (7.28)	78.31 (5.40)
**Gender, n (%)**				
	Female	20 (65)	126 (62.1)	18 (50)
	Male	11 (36)	77 (37.9)	18 (50)
Attitude toward technology (max=6), mean (SD)		4.92 (1.01)	4.61 (0.93)	3.89 (1.14)
Attitude toward aging (max=6), mean (SD)		3.91 (0.89)	4.16 (0.65)	3.89 (0.80)
Functional independence (max=7), mean (SD)		6.99 (0.36)	6.88 (0.65)	6.64 (0.84)

**Table 4:** Descriptive profiles of aging groups with positive and negative attitudes.

User factors		Positive aging attitudes (n=214)	Negative aging attitudes (n=56)
Age (years), mean (SD)		54.46 (13.25)	55.61 (19.27)
**Gender, n (%)**			
	Female	133 (62.1)	31 (55)
	Male	81 (37.9)	25 (45)
Attitudes toward technology (max=6), mean (SD)		4.62 (0.90)	4.29 (1.29)
Attitudes toward aging (max=6), mean (SD)		4.35 (0.54)	3.13 (0.34)
Functional independence (max=7), mean (SD)		6.91 (0.57)	6.68 (0.86)

## Results

### Data Analysis

Next to descriptive analyses, inferential statistics were conducted by means of multivariate analysis of variance analyses (MANOVA) to analyze the impact of the factors function and room on the acceptance of ultrasonic whistles in home care as well as effects of user diversity. For analysis of age, we used the three age groups as independent variables. Concerning attitudes toward aging, two groups were formed as independent variables based on scale values, provided that means larger than the scale center (mean>3.5) were grouped as positive attitudes, whereas means smaller than the scale center (mean<3.5) were classified as negative attitudes toward aging. In this case, different group sizes were accepted to separate people with a more optimistic age attitude from people with a more pessimistic age attitude.

The level of significance (*P* value) was set at 5%. As aging is naturally heterogeneous, we reported findings within the less restrictive level as marginally significant. Post hoc comparisons were done by Bonferroni correction. Mauchly test for sphericity was performed. In addition, the Huynh-Feldt adjustment was used to correct violation of sphericity. As for effect sizes, the partial eta-squared (η^2^) was reported.

The description of the Results section is structured as follows: first, the overall assessment of ultrasonic whistles in home care is presented descriptively. Next, its acceptance by function and room is considered in detail. In addition, user diversity effects are outlined.

### Users’ Assessment of Ultrasonic Whistles in Home Care

**Figure 4:**
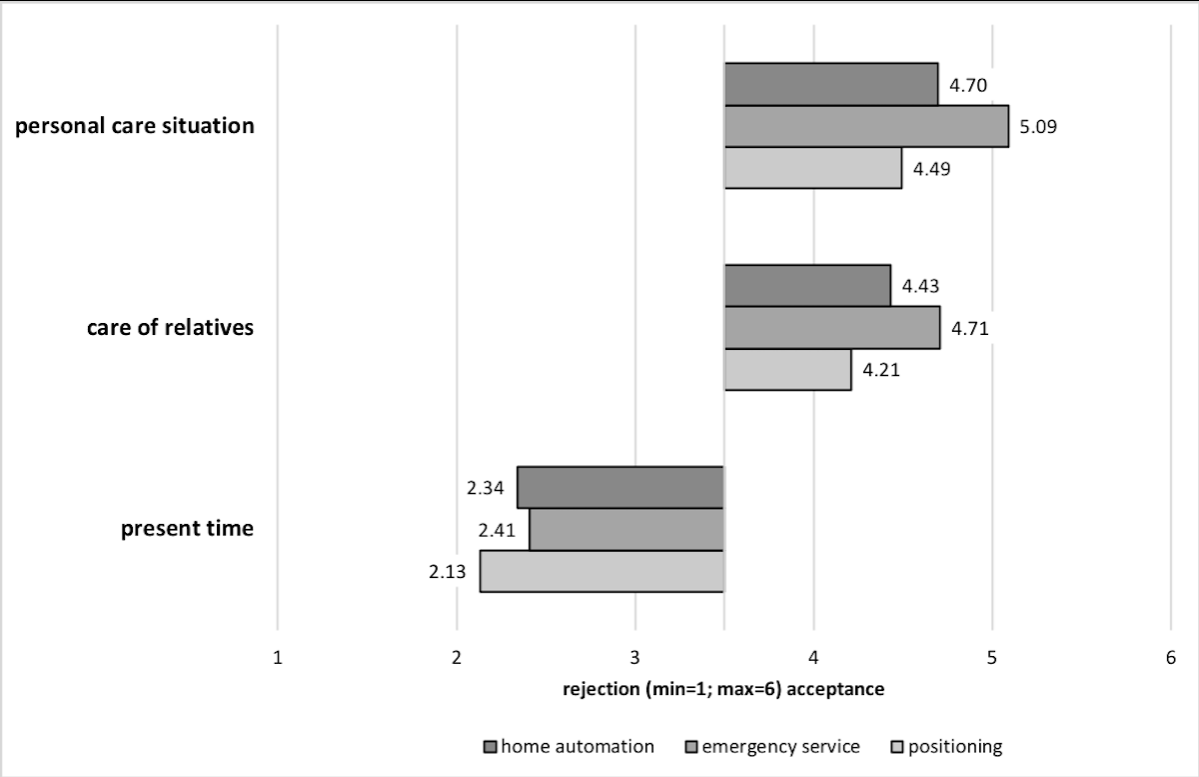
The diagram shows the evaluation of ultrasonic whistles referring to usage situations in relation to functional purposes (means).

In general, the assessment of ultrasonic whistles in home care was positive (mean 4.09 [SD 1.25]). Considering different usage situations, the use of ultrasonic whistles was generally accepted concerning *personal care situations* (mean 4.90 [SD 1.07]) and the *care of relatives* (mean 4.53 [SD 1.32]), whereas it was rejected to be used at the *present time* (mean 2.09 [SD 1.40]). In detail, this pattern was likely to occur with regard to specific functions in question, provided that emergency services were preferred in all cases, followed by home automation and positioning (see Figure 4).

To examine whether user groups differ in their perception and evaluation of different usage situations (dependent variables), MANOVA analyses were conducted with age, gender, and attitudes toward aging as independent variables. Results revealed significant main effects of age (*F*_18,502_=2.930; *P*<.001; η^2^=0.095) and gender (*F*_9,250_=2.072; *P*<.05; η^2^=0.069). In contrast, the attitudes toward aging did not impact the willingness to use the whistles in either of the contexts under study. In detail, age influenced the assessment of *emergency service* *at the present time* (*F*_2,258_=5.990; *P*<.01; η^2^=0.044), provided that elderly people (mean 3.11 [SD 1.79]) were more willing to adopt ultrasonic whistles for emergency situations immediately than the middle-aged group (mean 2.3 [SD 1.52]) and the younger group (mean 2.3 [SD 1.79]).

In addition, age gained an impact on the assessment of *positioning at the present time* (*F*_2,258_=3.788; *P*<.05; η^2^=0.029). This indicated that older adults tended to give positive evaluations in this context in comparison with both other age groups (older adults: mean 2.52 [SD 1.52]; middle-aged adults: mean 2.11 [SD 1.34]; and younger adults: mean 1.74 [SD 1.43]).

**Figure 5:**
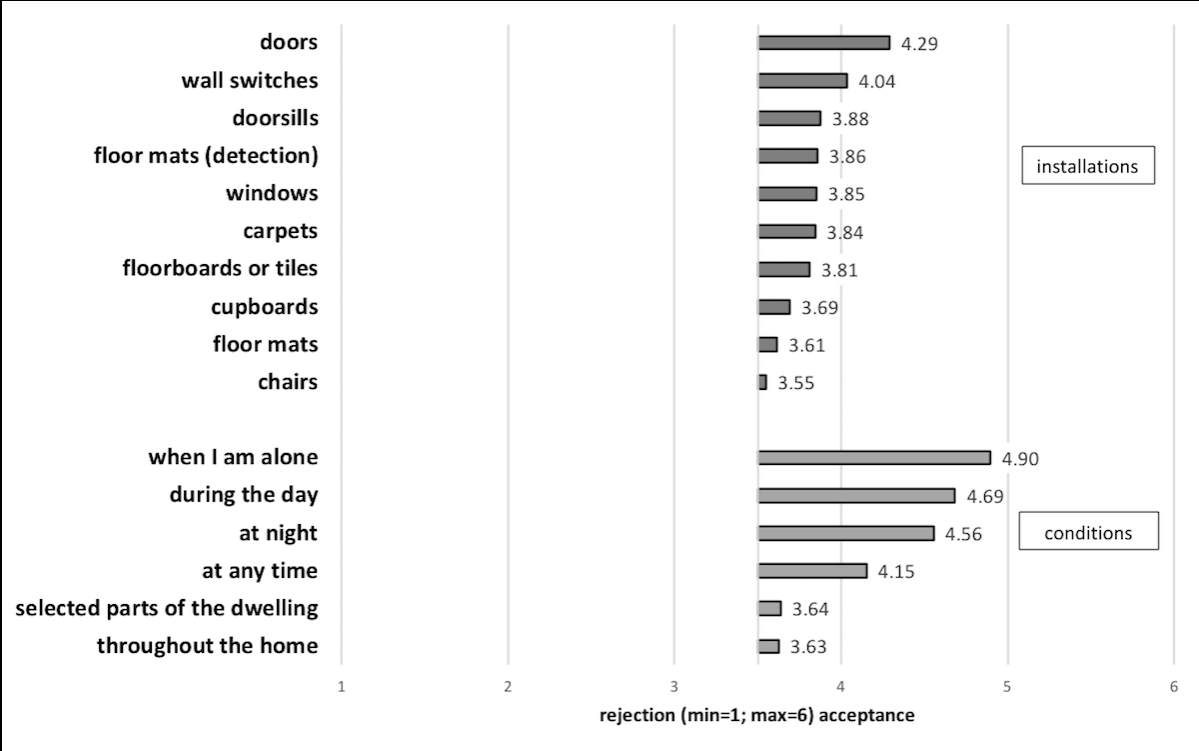
The diagram shows the assessment of specific installations and conditions referring to ultrasonic whistles in home care (means).

In general, Figure 5 shows that installations at *doors*, for example, for home automation, were valued the most (mean 4.29 [SD 1.49]), followed by *wall switches* (mean 4.04 [SD 1.52]), *windows* (mean 3.85 [SD 1.55]), *cupboards* (mean 3.69 [SD 1.56]), *floor mats* (mean 3.61 [SD 1.62]), and *chairs* (mean 3.55 [SD 1.55]). On average, the willingness to embed ultrasonic whistles in floors for fall detection was rather low (mean 3.78 [SD 1.41]). In this context, installations in *doorsills* (mean 3.88 [SD 1.64]) were rather more accepted than in *floor mats (detection;* mean 3.86 [SD 1.66]), *carpets* (mean 3.84 [SD 1.63]), and *floorboards or tiles* (mean 3.81 [SD 1.63]). Referring to specific conditions, the use of ultrasonic whistles in emergency call wristbands was most likely accepted (mean 4.57 [SD 1.26]), particularly concerning situations *when I am alone* (mean 4.9 [SD 1.3]) and *during the day* (mean 4.69 [SD 1.36]) compared with *at night* (mean 4.56 [SD 1.42]) and *at any time* (mean 4.15 [SD 1.56]). Moreover, assessments indicated that the acceptance of positioning systems, such as for fall detection, was slightly higher with regard to *selected parts of the dwelling* (mean 3.64 [SD 1.63]) than *throughout the home* (mean 3.63 [SD 1.65]).

For analyzing user diversity effects on the acceptance of specific installations and conditions as dependent variables, MANOVA analyses were conducted with age, gender, and attitudes toward aging as independent variables. For installations, analyses revealed a significant main effect of age as well as a significant interaction effect of age and attitudes toward aging (see Table 5). Assessments of installations options, for instance doors, were seen rather positive with regard to middle-aged (mean 4.42 [SD 1.37]) compared with young (mean 4.26 [SD 1.67]) and elderly users (mean 3.61 [SD 1.79]). For conditions, no significant omnibus effects were found (see Table 5).

### Users’ Acceptance by Function and Room

**Figure 6:**
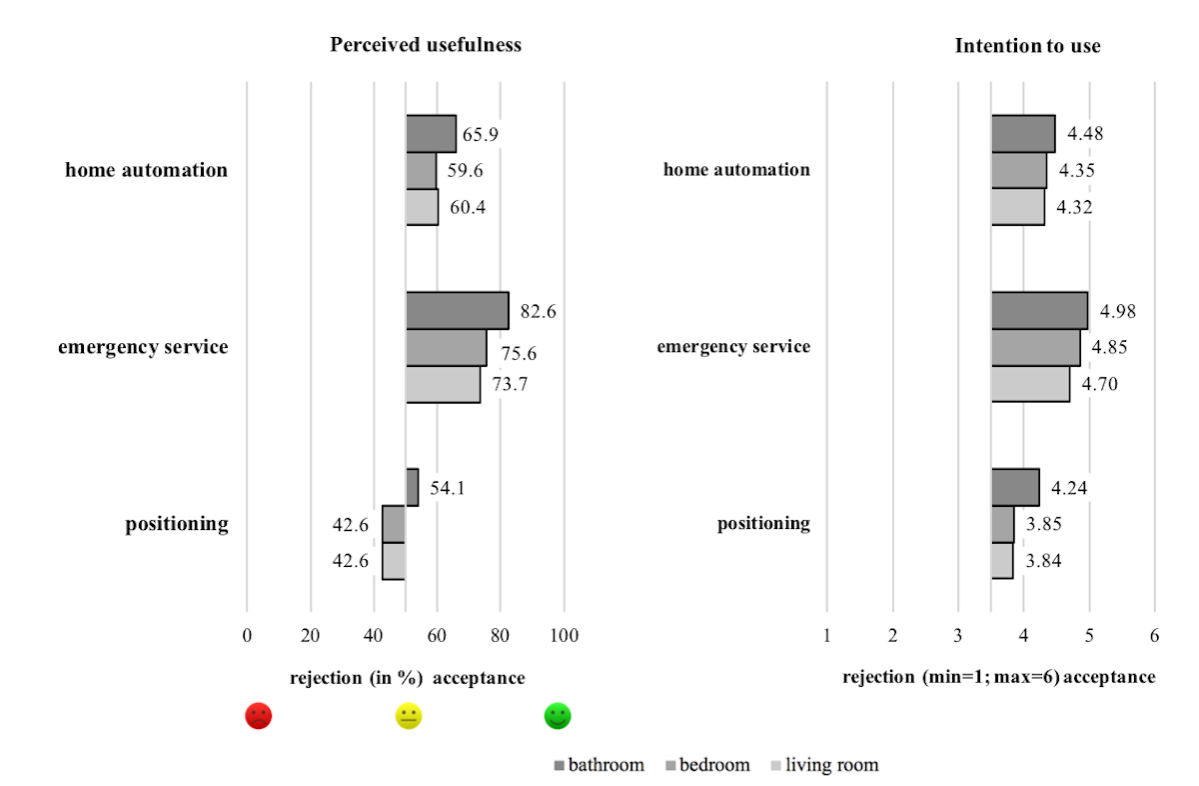
The diagrams show the perceived usefulness (left) and use intention (right) of ultrasonic whistles referring to function and room. For both scales, we used different response options. For the perceived usefulness, we used symbolic answering patterns (smileys, see bottom left), where participants gave their confirmation and rejection by percentages. For the use intention, we used 6-point Likert scales (min=1 “strongly disagree” and max=6 “strongly agree”), where mean values indicated acceptance or rejection (see bottom right).

Overall, the acceptance of ultrasonic whistles in home care was measured by its perceived usefulness and use intention referring to function and room. With regard to the perceived usefulness, ultrasonic whistles were commonly supported, particularly to be used in the bathroom (mean 73%, 197/270), followed by bedroom (mean 66.7%, 180/270) and living room (mean 63.3%, 171/270). In addition, taking into account specific functions, assessment results showed mean values above average (mean>50%), except for the use of positioning systems in bedroom and living room (mean 42.6%, 115/270; see Figure 6, left). However, with regard to the use intention, all applications in question were accepted (mean>3.5) though (see Figure 6, right). In total, the acceptance of emergency service reached peak levels across room boundaries, particularly in the bathroom (mean 4.98 [SD 1.24]). Slight restrictions were observable with regard to positioning in bedroom (mean 3.85 [SD 1.67]) and living room (mean 3.84 [SD 1.65]).

**Table 5:** Multivariate analysis of variance analyses results with age, attitudes toward aging, and gender as independent variables and installations and conditions as dependent variables.

User factors	Installations			Conditions	
	*F* test (*df*)	*P* value	η^2^	*F* test (*df*)	*P* value
Age	1.988 (20,500)	<.01	0.074	1.060 (12,508)	.39
Gender	1.308 (10,249)	.23	—^a^	1.949 (6,253)	.07
ATA^b^	0.660 (10,249)	.76	—	1.117 (6,253)	.35
Age × gender	0.942 (20,500)	.53	—	0.839 (12,508)	.61
Age × ATA	1.859 (20,500)	<.05	0.069	1.250 (12,508)	.25
Gender × ATA	1.559 (10,249)	.12	—	1.114 (6,253)	.36
Age × gender × ATA	1.017 (20,500)	.44	—	0.923 (12,508)	.52

^a^Not applicable.

^b^ATA: attitudes toward aging.

To test statistical significance of the factors function and room on the acceptance of ultrasonic whistles, a repeated measures analysis of variance was conducted. Overall, measurements revealed a significant main effect of function (*F*_2,269_=60.444; *P*<.001; η^2=^0.183) and room (*F*_2,269_=41.388; *P*<.001; η^2^=0.133) as well as a significant interaction effect of both factors (*F*_4,269_=8.701; *P*<.001; η^2^=0.031). According to effect sizes, the factor function gained the highest impact on the acceptance. In detail, emergency service was valued the most (mean 4.84 [SE 0.08]), followed by home automation (mean 4.38 [SE 0.09]) and positioning (mean 3.98 [SE 0.10]). Considering the factor room, the use of ultrasonic whistles was most likely accepted in the bathroom (mean 4.57 [SE 0.08]), followed by bedroom (mean 4.35 [SE 0.08]) and living room (mean 4.29 [SE 0.08]). With regard to the interaction of both factors, function and room, the use of emergency services in bathrooms influenced the acceptance of ultrasonic whistles in home care the most (see Figure 6, right).

As the factors function and room gain a significant influence on the acceptance of ultrasonic whistles in home care, it is of great interest whether and to what extent assessments differ with regard to diverse user groups, especially as the use of AAL technologies affects sensitive areas of life and, thus, is highly dependent on individual attitudes, demands, and concerns.

To compare the effect of user characteristics on the acceptance of ultrasonic whistles with regard to different user groups, MANOVA analyses were conducted. Age and attitudes toward aging were taken as independent variables and acceptance as dependent variable. Overall, no age effects were observable. By contrast, analyses showed a significant impact of the factor attitude toward aging (*F*_3,262_=3.098; *P*<.05; η^2^=0.034) as well as an interaction effect of age and aging (*F*_6,526_=2.383; *P*<.05; η^2^=0.026) on the acceptance. According to this, the acceptance of home automation was related to attitude toward aging (*F*_1,264_=3.214; *P*<.1; η^2^=0.012). In more detail, users with negative attitudes (mean 4.55 [SD 1.39]) accepted ultrasonic whistles more strongly than users with a positive attitude toward aging (mean 4.34 [SD 1.49]); however, these results were significant on the less restrictive level of 10 (*P*<.10).

## Discussion

### Principal Findings

This study aimed to reveal deeper insights into the user-centered assessment of ultrasonic whistles in home care, with special attention to age and attitude toward aging as AAL installations have a huge potential in the support for elderly care at home. Ultrasonic whistles have the advantage to be installable in existing home environments, thus representing an unobtrusive form of technology support at home. Our main focus was laid on the questions, for what reasons ultrasonic whistles would be perceived as useful at home (RQ1) and at which locations persons would accept ultrasonic whistles (RQ2). In addition, we explored whether user diversity factors, in particular, age and attitudes toward aging affect the assessment of ultrasonic whistles (RQ3).

### Acceptance of Ultrasonic Whistles

Overall, the assessment of ultrasonic whistles in home care is positive. In line with previous research, which shows that technology is accepted when the wish of older adults to age in place is respected [8,52] and the technology is perceived as useful [53,54], participants perceived ultrasonic whistles for home automation and for emergency services as highly useful and, as a consequence, reported to have a positive use intention for both contexts. Regarding the use of ultrasonic whistles for positioning, in contrast, the picture was more ambiguous. Participants’ evaluated the usefulness of ultrasonic whistles for positioning not that positive, but still they reported their willingness to use it if necessary (although to a lesser extent compared with home automation and emergency; RQ1).

Considering different usage situations, the use of ultrasonic whistles is accepted referring to personal care situations and the care of relatives, whereas it is rejected to be used at the present time. Apparently, the perceived usefulness of AAL systems is limited to the specificity of the caring situation. Participants did not appreciate using the technology at that time, presumably because the majority was in overall good health status and a high functional independence. However, this could also possibly be a kind of optimism bias, a psychological phenomenon, according to which individuals believe that they themselves are less vulnerable and less at risk in comparison with other people [55,56].

Confirming previous research on AAL acceptance [16,17], this study’s outcomes validate an influence of the factors function and room (RQ2), revealing that the use of ultrasonic whistles as for emergency service in bathroom is commonly preferred, followed by home automation and positioning in bedroom and living room. In contrast, Himmel and Ziefle [16] demonstrated that AAL acceptance decreases from rather public to private domestic spaces, provided that visual monitoring is accepted least and positioning best. However, in the mentioned paper, AAL acceptance was examined generically, without specifying a particular technology. It might be reasonably assumed that other applications in question as well as the range of functional description cause different assessment results in this context. For example, considering the evaluation of abstract monitoring systems in Himmel and Ziefle’s study [16], contrasting auditive, visual, and positioning technologies that have not been further specified, the acceptance of positioning turns out to be rather positive, whereas it is comparatively low when it comes to assessing a concrete technology such as the use of ultrasonic whistles for fall detection.

Furthermore, the outcomes of this study corroborate that the willingness to use ultrasonic whistles in home care is related to its perceived necessity along with commonly high safety needs, especially with regard to the broad acceptance of emergency services in bathrooms. This shows that older adults are not innovation-averse for age-related technology in general, but rather accept AAL technology that is perceived as useful and improves the quality of their daily life [52,53]. This was also confirmed by the findings in this study. As there is a general wish for aging in place [8,54], it is fair to assume that trade-offs between acceptance-related barriers, such as privacy concerns in intimate spaces, fall in favor of security and risk prevention as well as the opportunity to live independently and self-determined for as long as possible.

With regard to the user’s perspective (RQ3), our study results reveal that user diversity plays a minor role in the acceptance of ultrasonic whistles in home care. Although, a trend can be seen that the assessment of single functions differs among user groups, predominantly depending on age and attitudes toward aging which confirms previous studies [13,44]. According to this, the willingness to use home automation is higher by users with negative aging concepts who tend to fear restrictions on health, autonomy, and social life. Hence, there is reason to believe that ultrasonic whistles address heterogeneous user groups, particularly depending on its multifunctionality related to individual attitudes, demands, and concerns.

### Methodology

In this research, we used questionnaires to reach a broader sample of participants, both online and as paper-pencil version for those older adults who are not used to using the computer and internet. As participation was voluntary and not gratified, we can rely on a sample that was interested to participate, and according to some comments at the end of the questionnaire, participants were really motivated to contribute their opinions and expressed a high thematic involvement. Although one can assume honest and deliberate answering patterns by participants, one could critically argue that the sample reflects a group of symptomatic volunteers in terms of good aging, a comparably good health status, and high functional independence.

Having said that, we, thus, cannot exclude that the picture here reflects a kind of best case scenario, with a possible overestimation with respect to the acceptance of technology prevailing in a less biased sample. Future studies should, therefore, direct to including persons with a lesser health status, a more negative aging attitude, and with a lower level of functional interdependence to cover the full picture of aging and AAL technology acceptance.

Another aspect that should be critically considered is assessing the acceptance in scenario-based questionnaires reflects users’ acceptance attitudes, which should not automatically be equated to the willingness to use the technology in the end. This might be because of the fact that the vision of using a technology might be impacted by different constraints, circumstances, and takes place at different points in time in comparison with the real use in context. This is of particular impact, as potential usage barriers and perceived benefits can only be reliably assessed if users can actively interact with the ambient environment and *feel* the impact of the ultrasonic whistles in the real context. However, we cannot exclude that users might over- or underemphasize the potentials and pitfalls of ultrasonic whistles if their judgments only rely on the imagination of using it [36,57].

To achieve an overall understanding of the user’s acceptance in this context, further research is needed, particularly with regard to diverse user groups as well as perceived barriers and benefits. Only then it is possible to establish the potential for an accepted technology.

### Future Research

In total, the study revealed profound insights into the user-centered assessment of ultrasonic whistles in home care. However, the approach opens up a number of future research duties to complement the understanding of aging in place.

#### Validation in Real Ambient Assisted Living-Settings

In particular, the comparison of cross-study results revealed assessment shifts concerning the acceptance of AAL technologies in home care. Hence, in line with Wilkowska et al [36], an interconnection between research methods and objects is presumably leading to the conclusion that some factors are more context sensitive than others. On the basis of the assumption that the user-centered assessment of ultrasonic whistles may differ with regard to diverse research models, a multimethod approach is needed, particularly focusing on experimental study designs such as real-life smart home environments. Only then it is possible to enhance user experience by allowing participants to transform their ideas and concepts into reality. In future studies, we will, therefore, investigate in real-life environments (care institutions and private home environments) to what extent hands-on experiences with diverse applications enabled by the implementation of ultrasonic whistles change the perception and acceptance of ultrasonic whistles in home care.

#### Sample Composition

Another point that needs further research is the understanding of user diversity. Although we placed great emphasis on diverse target groups, such as age and health status, the study participants did not represent the group of the very elderly persons, who are definitely a major user group of AAL technologies. In addition, personal experience with AAL technologies was limited to single applications, predominately blood pressure monitors, as the overall health status was rather good. Thus, key findings should be validated, especially considering elderly users with health impairments and disabilities as well as diverse user roles (caregivers vs patients). Furthermore, it would be interesting to address participants of younger age groups as future users to explore individual requirements concerning the use of AAL technologies, using the example of ultrasonic whistles, which may differ because of generational background.

#### Intercultural Perspectives

Finally, the results of this study are shaped by culture- and country-specific norms, values, and standards of Germany. Beyond that, it would be insightful to compare assessment results against different cultural backgrounds with special attention given to diverse health care systems to contribute to a better understanding of acceptance-related factors in this context. In addition, especially the aging concepts and the value of aging varies across countries, social economies, and political systems [58]. As a consequence, also the extent of willingness of aged persons toward assistive technologies in the AAL context might vary. An intercultural picture of aging in place in combination with the study of the role and the functionality of AAL technologies is of paramount importance, not only for understanding the intercultural aging in place but also to inform technical designers, media, and policy about a responsible research and innovation, and last but not least, a public information strategy.

## Abbreviations

AAL: ambient assisted living
ATA: attitudes toward aging
ATT: attitude toward technology
MANOVA: multivariate analysis of variance analyses
RQ: research question
